# GBA1 Gene-Associated Transcriptomic Signatures Reveal Risk Genes in Parkinson’s Disease

**DOI:** 10.3390/biomedicines13112799

**Published:** 2025-11-17

**Authors:** Yanjun Liu, Xi Luo, Ronan M. T. Fleming

**Affiliations:** 1 School of Medicine, University of Galway, H91 TK33 Galway, Ireland; y.liu15@universityofgalway.ie (Y.L.); x.luo2@universityofgalway.ie (X.L.); 2 Digital Metabolic Twin Centre, University of Galway, H91 TK33 Galway, Ireland

**Keywords:** *GBA1*, Parkinson’s disease, Gaucher disease, neurodegeneration, Mendelian randomisation, genetic risk, pathogenesis

## Abstract

**Background/Objectives:** Pathogenic variants in the *GBA1* gene, which encodes the lysosomal enzyme β-glucocerebrosidase, cause Gaucher disease (GD) and represent one of the strongest genetic risk factors for Parkinson’s disease (PD). However, not all carriers develop PD, suggesting the involvement of additional modifiers. Transcriptomic alterations shared between GD and PD may reveal such modifiers and provide insights into the mechanisms linking *GBA1* to PD. **Methods:** Eighteen transcriptomic datasets spanning GD, *GBA1*-associated PD, and sporadic PD were integrated to identify shared, directionally concordant differentially expressed genes, followed by pathway enrichment analysis. Causal relationships were assessed using two-sample Mendelian randomisation with whole-blood and brain genetic instruments and PD GWAS summary statistics. Diagnostic relevance was evaluated in independent datasets using machine learning, while metabolic implications were explored with a neuron-specific genome-scale metabolic model. **Results:** Shared DEGs were enriched in lysosomal, lipid, redox, and endocrine pathways. Mendelian randomisation prioritised 12 risk genes in whole blood and 5 in brain tissue, with 4 overlapping; risk-increasing effects were observed for *GPNMB*, *MMP9*, *TRIM22*, *TESMIN*, *NFE2L3*, *FAM89A*, *METTL7A*, *PID1*, *NECAB2*, and *LPL*, whereas *GIPR* and *RASGRF2* showed protective effects, and *AGT* was brain-specific. Diagnostic signals were concentrated in a subset of genes, while metabolic modelling revealed convergent but subtype-specific perturbations across metabolic circuits. **Conclusions:** Convergent genetic, transcriptomic, and metabolic evidence supports at least two mechanistic routes to PD risk: a *GBA1*-sensitised lysosomal–lipid/redox axis, and a *GBA1*-independent neuronal–endocrine axis. These findings explain the variable risk among *GBA1* carriers, identify candidate biomarkers, and highlight pathway-anchored targets for stratified intervention.

## 1. Introduction

Gaucher disease (GD) is an autosomal recessive lysosomal storage disorder caused by biallelic mutations in the *GBA1* gene, which encodes the enzyme β-glucocerebrosidase. Loss of β-glucocerebrosidase activity impairs glycolipid degradation, leading to lysosomal accumulation of glucosylceramide and its deacylated derivative, glucosylsphingosine [1,2]. This accumulation disrupts lysosomal function and contributes to cellular dysfunction across multiple organ systems [3,4,5]. In neuronopathic forms of GD, glucosylceramide and glucosphingosine accumulate in the central nervous system, where they are directly neurotoxic and provoke neuroinflammatory responses, leading to neuronal injury and degeneration [5,6,7].

Beyond its classical definition as a rare metabolic disorder, GD is increasingly recognised for its connection to neurodegeneration, particularly Parkinson’s disease (PD) [8,9,10,11,12,13]. PD, the second most common neurodegenerative disorder, affects over 3% of individuals aged over 65 and is characterised by the progressive loss of dopaminergic neurons in the substantia nigra pars compacta and the presence of α-synuclein aggregates (Lewy bodies) [14]. Although advancing age is the greatest risk factor, both genetic and environmental contributors shape PD’s risk, onset, and progression [14,15]. Over 20 genes and more than 90 risk loci have been implicated in PD’s aetiology [14].

Among genetic contributors, heterozygous *GBA1* mutations represent the most common genetic risk factor for PD. Both GD patients and heterozygous carriers show an elevated lifetime risk of developing Parkinsonian features [8,9,10,11,12,13]. Pathogenic *GBA1* variants are present in 9.4% to 15% of PD patients across ethnic groups, with particularly high frequencies among Ashkenazi Jewish populations with sporadic PD [10,11,12,13]. *GBA1*-associated PD patients tend to present at a younger age, have more affected relatives, and exhibit atypical clinical features [10,11,12,13].

Mechanistically, reduced glucocerebrosidase activity in both *GBA1*-associated and sporadic PD patients mirrors the lysosomal dysfunction observed in GD [16,17,18]. The deficiency of glucocerebrosidase impairs the clearance of α-synuclein, a central protein in PD’s pathogenesis, promoting its aggregation into Lewy bodies, synaptic dysfunction, and neurotoxicity [19,20,21,22]. Additionally, mutant glucocerebrosidase can misfold and become retained in the endoplasmic reticulum, triggering endoplasmic reticulum stress and the unfolded protein response [16,23,24]. Broader disruptions include defective lipid trafficking, calcium dysregulation, and mitochondrial oxidative stress [3,25,26]. Synergistically, glucosylceramide and α-synuclein have been shown to activate microglia and fuel neuroinflammation, paralleling inflammatory cascades in GD [27]. These findings suggest a shared pathogenic axis between GD and PD involving lysosomal–autophagic dysfunction, lipid dysregulation, protein aggregation, and chronic neuroinflammation [9,20,24].

Despite this established association, *GBA1* mutations exhibit incomplete penetrance, meaning that not all mutation carriers develop PD. By age 80, the risk of PD is 9.1% in GD patients and 7.7% in heterozygotes, compared to 2.1% in non-carriers [8]. Notably, a 10-year follow-up of nine individuals with high-risk L444P/+ variants reported no PD development [28]. These observations indicate that genetic background, environmental, epigenetic, and other factors are also important determinants of disease onset and progression.

In light of these insights, we hypothesised that transcriptomic changes observed in GD may precede or contribute to PD’s pathogenesis, potentially revealing early molecular signatures indicative of PD risk. To test this, we integrated transcriptomic data from GD and PD, assessed the causal relevance of shared expression changes using Mendelian randomisation, examined their functional roles through metabolic modelling, and evaluated their diagnostic value with machine learning in independent cohorts. 

## 2. Materials and Methods

**Figure 1:**
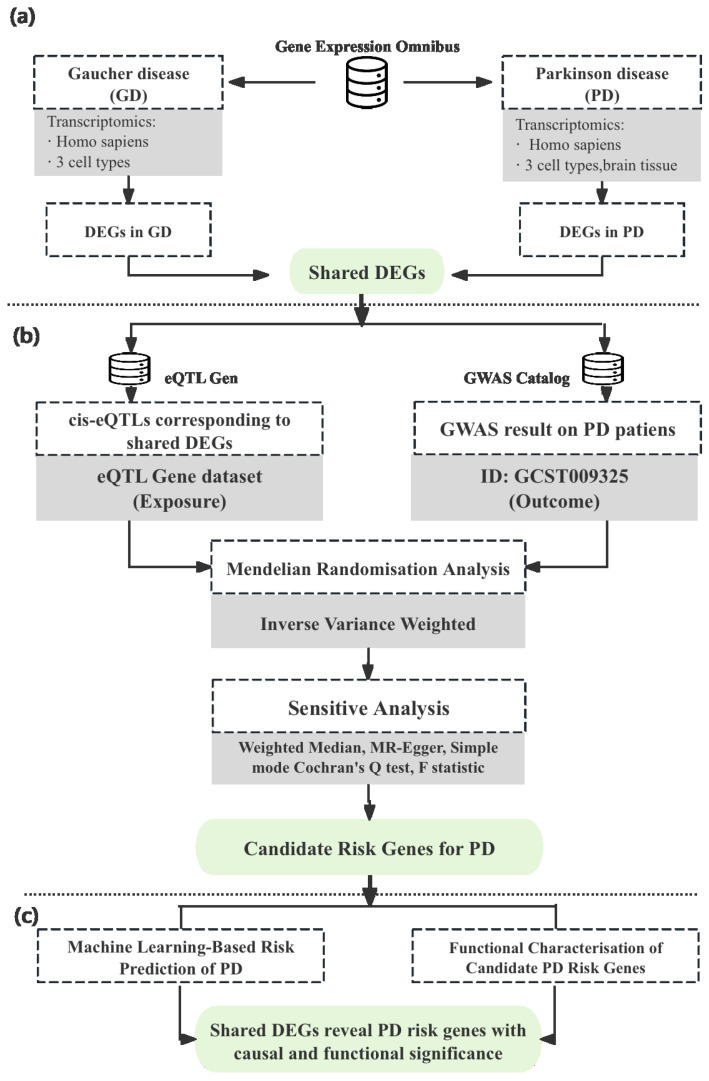
Integrative workflow for identification and characterisation of candidate risk genes for Parkinson’s disease. (**a**) Differentially expressed genes (DEGs) in Gaucher disease (GD) and Parkinson’s disease (PD) were obtained from transcriptomic datasets in the Gene Expression Omnibus. Shared DEGs across GD and PD were identified as potential molecular links. (**b**) Mendelian randomisation (MR) analysis was performed using cis-eQTLs for the shared DEGs (from whole blood [29] and brain [30]) as exposures and GWAS summary statistics for PD (GCST009325 [31]) as outcomes. The primary MR method used was inverse-variance weighted [31], supported by sensitivity analyses including MR-Egger [32], weighted median [33], and heterogeneity tests. (**c**) Functional evaluation of MR-prioritised PD risk genes by classifying PD cases versus controls using transcriptomic data, and assessing their metabolic impact in a dopaminergic-neuron metabolic model (iDopa model [34]).

An overview of the study design and analytical workflow is presented in Figure 1.

### 2.1. Identification of Shared DEGs in Gaucher and Parkinson’s Diseases

#### 2.1.1. Transcriptomic Data Selection

Raw gene expression data relevant to GD and PD (*GBA1*-associated PD and sporadic PD) were retrieved from the Gene Expression Omnibus database, encompassing both high-throughput sequencing and microarray-based transcriptomic profiles. The datasets included gene expression profiles from commonly used cellular models in GD and PD research, including neurons, fibroblasts, and macrophages, as well as post-mortem brain tissue. The inclusion of diverse sample types enabled the investigation of both systemic and tissue-specific transcriptomic alterations associated with GD and PD.

For GD, datasets were selected based on the following criteria: (i) human-derived transcriptomic data, and (ii) samples originating from cell types commonly affected in the disease, including macrophages, fibroblasts, and neurons. For PD, the inclusion criteria comprised (i) *GBA1*-associated PD cases, involving individuals with *GBA1* mutations and a clinical diagnosis of PD; (ii) sporadic PD cases lacking known genetic mutations; (iii) samples derived from macrophages, fibroblasts, neurons, or post-mortem brain tissue; and (iv) datasets published within the past five years. All datasets were screened for data quality, disease relevance, and the availability of sufficient metadata (e.g., disease classification and cell type) to ensure consistency in downstream analyses.

#### 2.1.2. Transcriptomic Data Analysis

For each selected dataset, differential gene expression analysis was conducted using the DESeq2 package (v1.46.0) [35] where raw count data were available. For datasets containing only normalised expression values (e.g., microarray data), the limma package (v3.62.2) [36] was applied. Prior to analysis, samples were annotated and filtered to retain only those representing clearly defined case (disease) and control (unaffected) groups. Lowly expressed genes were removed through gene filtering, and normalisation procedures were applied to ensure comparability across samples.

To mitigate batch effects and heterogeneity arising from differences in study design and experimental platforms, differential expression analyses were performed independently for each dataset, rather than on merged expression matrices. DEGs were identified using a false discovery rate (FDR) threshold of 0.05 and $log_{2}\left(foldChange\right)$ of at least 1. This dataset-specific approach ensured that cell- or tissue-specific transcriptional changes were captured while avoiding cross-platform artefacts. To enable cross-dataset harmonisation, only statistically significant DEGs and their direction of regulation were compared across studies. Shared DEGs were defined as genes that were significantly and consistently differentially expressed in at least two datasets per condition (GD, *GBA1*-associated PD, sporadic PD), with the same direction of regulation.

To confirm that shared DEGs exhibited consistent expression patterns across conditions, their expression trends were compared across all datasets. This analysis aimed to determine whether shared DEGs were similarly regulated in GD, *GBA1*-associated PD, and sporadic PD. Genes showing concordant expression changes across all three conditions were considered to be putative indicators of shared pathogenic mechanisms. For the subset of DEGs consistently altered across both GD and PD conditions, Gene Ontology (GO) pathway enrichment analysis was conducted to explore their associated biological functions and enriched physiological pathways.

### 2.2. Genetic Risk Association

To investigate the potential causal contribution of the shared DEGs to PD risk, we applied Mendelian randomisation analysis, a method well suited for inferring causality in the context of observational data. It is a statistical approach that uses genetic variants as instrumental variables to infer the causal effect of a modifiable exposure, such as gene expression, on an outcome, such as PD risk. Specifically, expression-associated variants were used to proxy for gene expression levels, thereby enabling estimation of the causal effect of gene expression on PD risk. In contrast to conventional association analyses, which are often limited by confounding factors and the potential for reverse causation, Mendelian randomisation reduces these biases because genetic variants are randomly allocated at conception [37,38]. This approach therefore provides evidence as to whether altered expression of the shared DEGs represents a causal factor in PD’s pathogenesis rather than a downstream consequence of the disease.

#### 2.2.1. Identification of cis-eQTL Instruments

Cis-expression quantitative trait loci (cis-eQTLs), defined as genetic variants located within ±1 Mb of a gene that influence its expression levels, were used as instrumental variables. Cis-eQTLs for the shared DEGs were obtained from whole blood [29] and brain tissue [30], providing a large-scale meta-analysis of eQTLs derived from human whole-blood and brain data. To ensure instrument strength and reduce confounding due to linkage disequilibrium, variants were filtered to include only those with genome-wide suggestive significance ($p<5\times 10^{-6}$), pairwise independence based on linkage disequilibrium pruning ($r^{2}<0.3$), and a minor allele frequency greater than 1%. The F-statistic (F = β^2^/SE^2^) was calculated for each cis-eQTL single-nucleotide polymorphism (SNP) to evaluate instrument strength, and only variants with F-statistic > 10 were retained for MR analyses to minimise potential weak-instrument bias.

The filtered cis-eQTL SNPs were then cross-referenced with summary statistics from a large-scale genome-wide association study (GWAS) of PD, which included 33,674 cases and 482,730 controls ([31]; GWAS Catalog accession GCST009325). To avoid strand ambiguity, palindromic SNPs with a minor allele frequency greater than 0.42 were excluded. Additionally, to reduce the risk of reverse causation, SNPs showing stronger association with PD ($p<5\times 10^{-6}$) than with gene expression were also excluded. The remaining variants served as instrumental variables in Mendelian randomisation analyses to estimate the causal effect of gene expression levels on PD risk.

#### 2.2.2. Mendelian Randomisation and Sensitive Analyses

Causal inference was conducted using four complementary methods: inverse-variance weighted [39], Egger regression [32], weighted median [33], and simple mode estimators [40]. Our primary estimate came from the inverse-variance weighted method—applying a fixed-effect model when Cochran’s Q test (p>0.05) indicated no heterogeneity, and switching to a random-effects inverse-variance weighted model when heterogeneity was detected (*p* < 0.05). Egger regression provided both a causal estimate and a test for directional pleiotropy via its intercept. Analyses were carried out in R (v4.4.1) with the TwoSampleMR package (v0.6.17) [41], and statistical significance was set at α=0.05 for all analyses.

### 2.3. Functional Analysis of Risk Genes

#### 2.3.1. Diagnostic Performance

To evaluate the predictive value of Mendelian randomisation-prioritised genes (hereafter, risk genes) for PD, two independent transcriptomic datasets were analysed: post-mortem brain tissue from sporadic PD and controls (GSE205450) [42], and induced pluripotent stem cell (iPSC)-derived midbrain organoids from healthy individuals and PD patients carrying the *GBA1* N370S mutation (GSE287566). Within each dataset, single-gene discrimination was assessed using univariable logistic regression, with performance summarised by the area under the receiver operating characteristic (ROC) curve (AUC). Ninety-five-percent confidence intervals (95% CIs) for AUCs were computed using the DeLong method [43]. The combined diagnostic capacity of all risk genes was modelled using LASSO (Least Absolute Shrinkage and Selection Operator) logistic regression [44], which performs variable selection by shrinking uninformative coefficients to zero. This approach was chosen to reduce overfitting when the number of predictors is high relative to the sample size. Diagnostic performance was estimated using nested, stratified k-fold cross-validation to prevent over-optimistic results from tuning on the same data used for evaluation. Performance was estimated using a two-stage cross-validation designed to avoid over-optimistic results. The dataset was divided into several equal parts. In each round, one part was set aside as a temporary test set, and the model was trained on the remaining parts. Within the training data only, a second round of cross-validation chose the regularisation strength using the conservative rule (i.e., the simplest model whose cross-validated performance was within one standard error of the best). The tuned model was then used to predict the set-aside test part. Predicted probabilities from all rounds were combined to produce a single ROC curve and AUC based entirely on samples not used for training. Statistical comparisons between single-gene and LASSO-model AUCs were performed using DeLong’s test for correlated ROC curves, with multiple-testing correction applied using the Benjamini–Hochberg method [43]. 

#### 2.3.2. Metabolic Modelling

To assess the metabolic impact of risk genes and compare subtype-specific effects, gene expression data from purified human iPSC-derived midbrain dopaminergic neurons were analysed (GSE62642 [45]), with samples classified as controls, sporadic PD, or *GBA1*-associated PD. Transcriptomic changes were interpreted using the iDopa model, an established, neuron-specific, genome-scale reconstruction of human induced dopaminergic neurons [34]. The model provides curated gene–protein–reaction rules and captures pathways central to dopaminergic neurobiology. Gene expression was mapped to model genes, and case–control differences were propagated to enzyme-catalysed reactions.

Differential expression was estimated for two contrasts: sporadic PD vs. controls, and *GBA1*-associated PD vs. controls. Gene-level changes were mapped to enzyme-catalysed reactions using the model’s gene–protein–reaction rules. Reporter metabolites were then identified with the reporter metabolites algorithm [46], which summarises the differential expression of enzymes that produce or consume each metabolite. Metabolites were ranked under false discovery rate (FDR) control. High-scoring metabolites were treated as local network “hotspots” likely to show altered concentrations. In parallel, reactions linked to differentially expressed genes were tested for enrichment within the model’s predefined subsystems. Results from the two contrasts were compared to distinguish changes shared by both PD subtypes from those specific to *GBA1*-associated or sporadic PD. 

## 3. Results

### 3.1. Identification of Shared Transcriptomic Alterations in Gaucher and Parkinson’s Diseases

Eighteen transcriptomic datasets were retrieved from the Gene Expression Omnibus database (Table 1) to investigate potential transcriptomic overlap between GD and PD (*GBA1*-associated PD and sporadic PD). These datasets encompass gene expression profiles from commonly utilised cellular models in GD and PD research, including neurons, fibroblasts, and macrophages, as well as post-mortem brain tissue. The integration of diverse sample types facilitated the assessment of both systemic and tissue-specific transcriptomic alterations associated with GD and PD.

**Figure 2:**
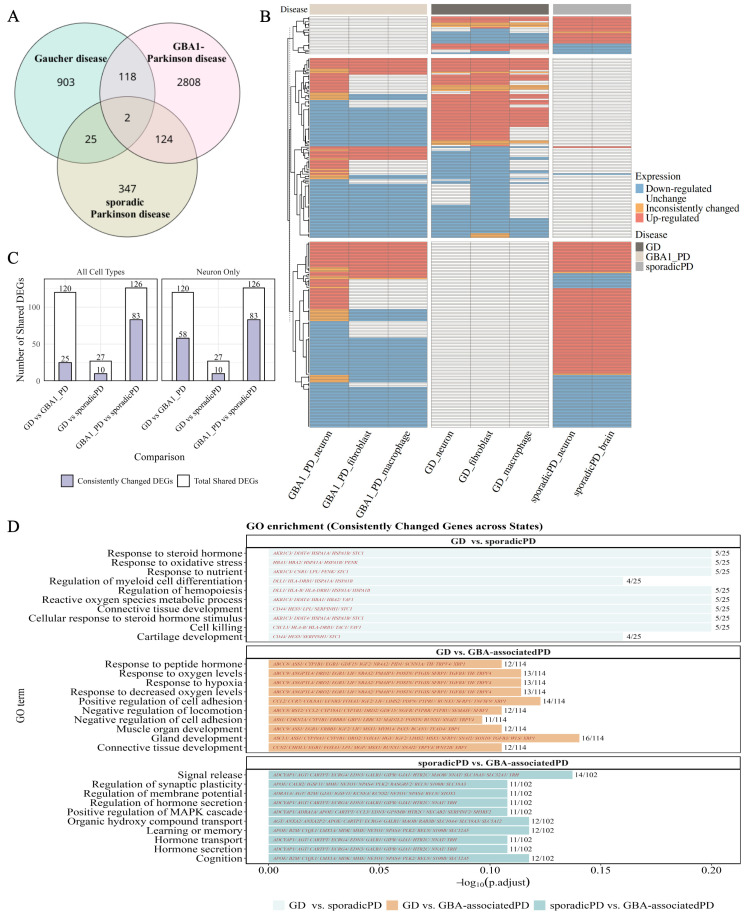
Shared transcriptomic alterations in Gaucher disease and Parkinson’s disease. (**A**) Shared DEGs between Gaucher disease, *GBA1*-associated PD, and sporadic PD. (**B**) Expression profiles of the shared DEGs across the three conditions. (**C**) Comparison and summary of shared DEGs with consistent expression patterns. (**D**) Gene Ontology enrichment analysis of consistently expressed shared DEGs. Enriched genes highlighted on the bars. Gene ratio (shown above each bar) was calculated as the number of genes from shared DEGs within a given pathway divided by the total number of total shared DEGs.

Across the GD datasets, a total of 1048 DEGs were identified, compared to 3052 in the *GBA1*-associated PD datasets and 498 in the sporadic PD datasets. Among these, 120 DEGs were shared between GD and *GBA1*-associated PD, 27 were shared between GD and sporadic PD, and 126 were shared between *GBA1*-associated PD and sporadic PD (Figure 2A), revealing potential common molecular signatures across the three conditions.

To assess the consistency of expression trends, these shared DEGs were further analysed (Figure 2B,C). A total of 25 genes exhibited concordant expression patterns between GD and *GBA1*-associated PD, 10 between GD and sporadic PD, and 83 between *GBA1*-associated PD and sporadic PD, across all cell types. Notably, when focusing exclusively on neuronal datasets, the number of shared DEGs between GD and *GBA1*-associated PD increased to 58. In neurons, the observed overlaps are likely to represent transcriptional changes that occur early in PD’s pathogenesis.

To explore the biological functions of DEGs with consistent expression patterns, pathway enrichment analysis was performed (Figure 2D). Among the DEGs shared between GD and *GBA1*-associated PD, pathways related to hypoxia and the response to hypoxia were significantly enriched, suggesting that alterations in oxidative phosphorylation may be a common functional hallmark in both conditions. For the comparison between GD and sporadic PD, enriched pathways included nutrient sensing—a key function of lysosomes—together with oxidative stress and hormone response, implicating lysosomal dysfunction, redox imbalance, and hormonal dysregulation as features shared independently of *GBA1* mutations. Between *GBA1*-associated and sporadic PD, pathways related to cognitive impairment, memory deficits, and hormone imbalance were enriched, highlighting common neurodegenerative and endocrine-related dysfunctions in both PD subtypes.

### 3.2. Causal Inference of Shared DEGs in Parkinson’s Disease

The observed molecular overlap between GD, *GBA1*-associated PD, and sporadic PD prompted us to investigate whether shared DEGs contribute directly to PD’s pathogenesis. Of the 269 shared DEGs (combined from the three pairwise comparisons), 73 possessed at least one genome-wide significant cis-eQTL within ±1 Mb, qualifying them for Mendelian randomisation analyses using PD GWAS summary statistics. In total, 5945 SNPs were included to evaluate associations between gene expression and PD risk. The number of instrumental SNPs per gene ranged from 2 to 415, with a median of 51 (Supplementary Table S1). 

**Figure 3:**
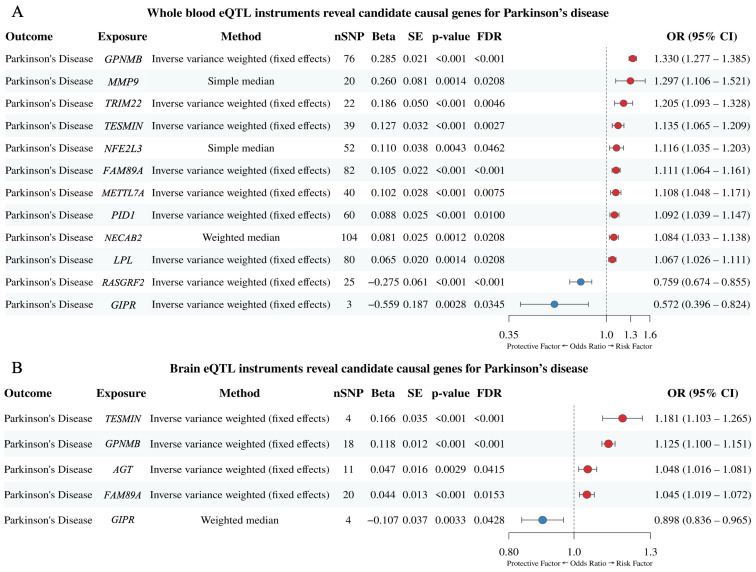
Genetic evidence supporting causal roles of shared DEGs in Parkinson’s disease risk. Forest plots of odds ratios (ORs) with 95% confidence intervals for genes showing significant causal associations with Parkinson’s disease (FDR < 0.05) using eQTLs from (**A**) whole blood and (**B**) brain tissue. Genes are ordered by effect size; red points indicate risk (OR > 1) and blue points indicate protection (OR < 1).

Using whole-blood cis-eQTL instruments, Mendelian randomisation identified 12 genes with statistically significant evidence of a causal effect on the odds of PD after FDR correction (FDR < 0.05; Figure 3A). The largest effects were observed for *GPNMB*, followed by *MMP9* and *TRIM22*, where a one-standard-deviation increase in genetically proxied expression corresponded to approximately 33%, 29.7%, and 20.5% higher odds of PD, respectively. Additional risk-increasing associations were seen for *TESMIN*, *NFE2L3*, *FAM89A* and *METTL7A*, with smaller effects for *PID1*, *NECAB2*, and *LPL*. In contrast, higher expression of *RASGRF2* and *GIPR* was associated with lower PD odds, consistent with protective effects.

Using brain cis-eQTL instruments, Mendelian randomisation identified five genes with statistically significant evidence of a causal effect on the odds of PD after FDR correction (FDR < 0.05; Figure 3B). Four of these signals overlapped with those detected in the whole-blood analysis, indicating tissue-robust effects. The remaining signal, *AGT*, was unique to the brain analysis, corresponding to an approximately 4.9% increase in PD risk per one-standard-deviation increase in genetically proxied expression.

To ensure instrument validity, only SNPs with F-statistic > 10 were retained for MR analyses, with the mean F-statistics per gene ranging from 18.4 to 95.2 (median = 42.6), confirming adequate instrument strength (Supplementary Tables S1 and S2). Cochran’s Q test showed no significant heterogeneity (all *p* > 0.05) (Supplementary Tables S3 and S4), and MR-Egger intercepts did not indicate directional pleiotropy (all *p* > 0.05), supporting the reliability of the causal estimates (Supplementary Tables S5 and S6). Sensitivity analyses using the weighted median, simple mode, and Egger regression methods yielded effect directions consistent with the primary inverse-variance weighted results, although some associations did not reach statistical significance (Supplementary Tables S7 and S8). Collectively, these analyses identified a subset of shared DEGs with potential causal involvement in PD’s pathogenesis, comprising 12 genes from whole blood and 5 from brain tissue (4 overlapping), hereafter referred to as risk genes.

### 3.3. Functional Characterisation of Risk Genes

#### 3.3.1. Diagnostic Performance of Risk Genes

**Figure 4:**
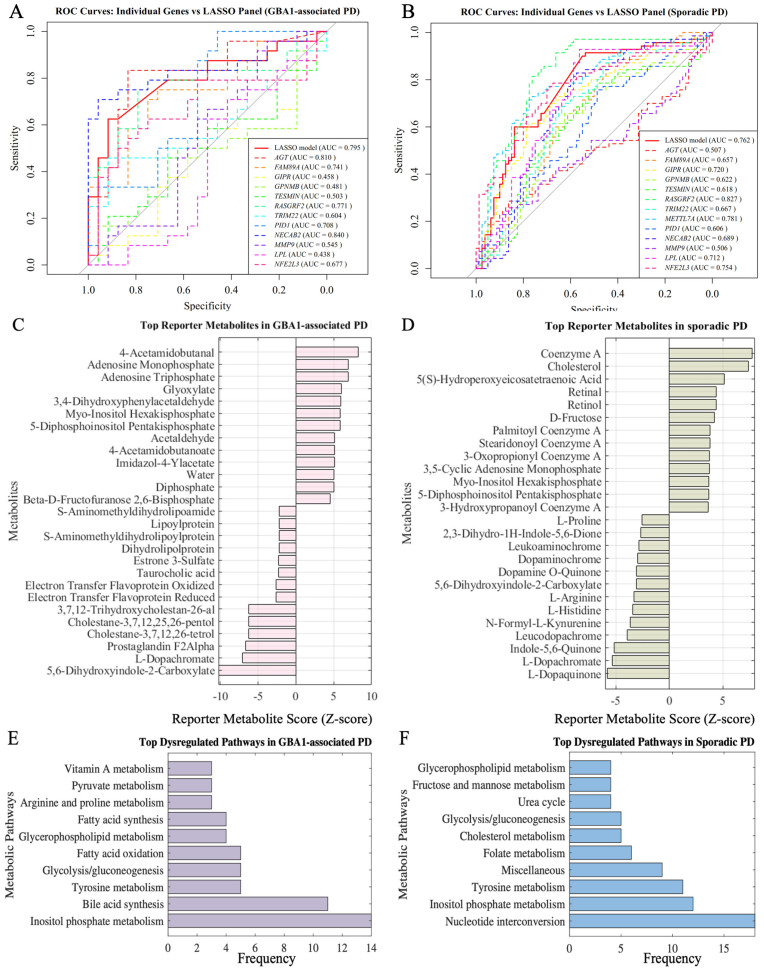
Functional characterisation of risk genes. (**A**,**B**) ROC curves comparing single-gene classifiers with a multivariable LASSO model using all candidate genes to distinguish (**A**) *GBA1*-associated PD and (**B**) sporadic PD from healthy controls. (**C**,**D**) Reporter metabolites predicted by the dopaminergic-neuron metabolic model for (**C**) *GBA1*-associated PD and (**D**) sporadic PD. (**E**,**F**) Dysregulated pathways predicted by the dopaminergic-neuron metabolic model for (**E**) *GBA1*-associated PD and (**F**) sporadic PD.

To evaluate the diagnostic value of Mendelian randomisation-prioritised genes (hereafter, risk genes), the discriminative capacity of each gene was assessed using univariate ROC analysis, and the combined effect of all genes was assessed using a LASSO logistic regression model in two independent transcriptomic datasets: *GBA1*-associated PD (N370S/+) and sporadic PD. In the *GBA1*-associated PD dataset, cross-validated ROC analysis of the pre-specified genes showed that several single markers already carried strong discriminatory signals (Figure 4A). The LASSO panel achieved an out-of-fold AUC = 0.795 (95% CI 0.664–0.927), while individual genes such as *NECAB2* (AUC = 0.84, 95% CI 0.718–0.963), *AGT* (0.81, 0.678–0.941), and *RASGRF2* (0.77, 0.630–0.912) performed best; others were moderate to weak (e.g., *PID1* ≈ 0.71, 0.557–0.860; *FAM89A* ≈ 0.74, 0.599–0.884; several near chance). DeLong’s test for correlated ROC curves revealed no statistically significant differences between any single-gene model and the LASSO panel (e.g., NECAB2: Δ AUC = 0.045, 95% CI –0.041 to 0.131, *p* = 0.30; AGT: Δ AUC = 0.015, 95% CI –0.074 to 0.103, *p* = 0.74).

In the sporadic PD dataset, the strongest individual marker was RASGRF2 (AUC = 0.827, 95% CI 0.758–0.896), followed by *METTL7A* (0.781, 0.706–0.857) and *NFE2L3* (0.754, 0.673–0.835). Moderate performance was observed for *GIPR* (0.720, 0.637–0.803) and *LPL* (0.712, 0.626–0.797) (Figure 4B). A cross-validated LASSO logistic model combining the full gene set achieved AUC = 0.762 (95% CI 0.684–0.840), outperforming most single genes but not the top markers (RASGRF2 and *METTL7A*). DeLong’s test for correlated ROC curves indicated that RASGRF2 significantly outperformed the LASSO panel (Δ AUC = 0.065, 95% CI 0.021–0.109, *p* = 0.0038, FDR = 0.0076), whereas METTL7A showed no significant difference (Δ AUC = 0.019, 95% CI 0.050–0.089, *p* = 0.58). No other single gene exceeded the LASSO model with statistical significance after multiple-testing correction. The lower AUC of the panel relative to the best single gene likely reflects limited incremental signal beyond the dominant marker and substantial redundancy among genes.

#### 3.3.2. Metabolic Impact of Risk Genes in Parkinson’s Disease Subtypes

To assess the metabolic impact of risk genes and compare subtype-specific effects, expression data from *GBA1*-associated PD and sporadic PD were interpreted using the iDopa model. Reporter-metabolite and pathway analyses revealed both shared and subtype-specific metabolic changes in dopaminergic neurons. Across PD subtypes, signals converged on inositol-phosphate metabolism, glycerophospholipid turnover, glycolysis/gluconeogenesis, and tyrosine/catecholamine chemistry, indicating pressure on membrane signalling, energy handling, and dopamine-related oxidation (Figure 4C–F).

In *GBA1*-associated PD, the top-ranked reporter metabolites clustered around lipid and energy metabolites (e.g., inositol pyrophosphates, adenine nucleotides, acyl-CoA-linked species, and glutathione disulphide), consistent with altered phosphoinositide signalling, membrane remodelling, and redox balance (Figure 4C). Pathway enrichment mirrored these findings, prioritising inositol-phosphate metabolism, glycolysis/gluconeogenesis, glycerophospholipid metabolism, and both fatty acid oxidation and fatty acid synthesis, with additional support for bile acid synthesis, tyrosine metabolism, and arginine/proline and pyruvate metabolism (Figure 4E).

In sporadic PD, reporter-metabolite hotspots again implicated inositol-phosphate and glycerophospholipid pathways, alongside glycolysis/gluconeogenesis and tyrosine-related chemistry. Additional prominent metabolites indicated involvement of cholesterol and CoA-linked intermediates (Figure 4D). Pathway analysis highlighted nucleotide interconversion and folate metabolism, together with cholesterol metabolism, the urea cycle, and fructose/mannose metabolism, alongside the shared inositol-phosphate, glycolytic, and glycerophospholipid pathways (Figure 4F). These analyses point to common metabolic pressure points in PD, including membrane phosphoinositide signalling, glycolytic flux, lipid membrane dynamics, and catecholamine handling, while suggesting a greater emphasis on fatty acid and bile acid pathways in *GBA1*-associated PD and on nucleotide, one-carbon, and sterol handling in sporadic PD.

## 4. Discussion

In this study, we integrated differential expression, Mendelian randomisation, and metabolic modelling to dissect shared molecular signatures linking GD, *GBA1*-associated PD, and sporadic PD. Comparative transcriptomic analysis identified overlapping DEGs across these conditions, while complementary Mendelian randomisation approaches prioritised a subset as putative causal contributors to PD risk. Integrating these results with independent transcriptomic datasets and genome-scale metabolic models of dopaminergic neurons highlighted both *GBA1*-dependent and *GBA1*-independent routes to PD risk.

The shared DEGs between GD and PD fell into two main categories: The first comprised genes that were shared between GD and *GBA1*-associated PD, or between GD and sporadic PD. These genes likely reflect common pathological pathways linking GD to PD, with their altered expression potentially initiated by *GBA1* mutations, enzyme deficiency, or other triggers. Because *GBA1*-related alterations typically occur before PD’s onset, these genes may represent transcriptional hubs that are particularly susceptible to perturbation, whether by *GBA1* dysfunction or by unrelated factors, as indicated by their presence in sporadic PD cases without *GBA1* mutations. Pathway analysis revealed that these genes are enriched in mitochondrial function, lysosomal activity, redox homeostasis, and hormonal regulation, all well-established mechanisms in PD’s pathogenesis [58]. The second category included DEGs shared between *GBA1*-associated PD and sporadic PD but not found in GD. These genes may contribute to PD risk independently of *GBA1* dysfunction. They were enriched in pathways related to cognitive impairment, memory deficits, and hormonal imbalance, suggesting that risk factors linked to higher-order cognitive functions may influence susceptibility to PD in both *GBA1* mutation carriers and the general population [59,60]. The robustness of these findings is supported by the repeated identification of these DEGs across multiple datasets, as well as by consistency with previous individual studies indicating that not only are mutations in the *GBA1* gene a contributor to the occurrence of PD, they also lead to a form of PD with more significant and rapid cognitive decline [61,62].

Mendelian randomisation analyses integrating PD GWAS and eQTL data revealed 12 candidate genes in whole blood and 5 in brain tissue, with 4 overlapping between tissues. Notably, several genes showed concordant effects across tissues, suggesting shared systemic influences, whereas others were specific to the brain, highlighting tissue-dependent mechanisms. Functional annotation indicated that the risk genes converge on pathways regulating lipid and cholesterol metabolism, glucose–insulin signalling, the balance between glycolysis and oxidative phosphorylation, oxidative stress responses, neuroinflammation, immune regulation, and neuronal signalling. Some genes—such as *GPNMB*, which encodes glycoprotein nonmetastatic melanoma protein B—have well-established roles in neurodegeneration, while others remain largely unexplored yet display plausible mechanistic connections based on their involvement in metabolic, inflammatory, or synaptic processes.

Among the four overlapping signals, *GPNMB* stands out as the most mature candidate gene. *GPNMB* is an endogenous type I transmembrane glycoprotein, first described in melanoma biology, that is elevated in multiple neurodegenerative conditions, including Alzheimer’s disease [63], amyotrophic lateral sclerosis [64], and PD [65]. Prior biomarker studies have consistently reported elevated *GPNMB* protein levels in the plasma and cerebrospinal fluid of GD [66,67] and Niemann–Pick type C patients [68], suggesting systemic lysosomal stress-related upregulation. Multiple PD GWASs map the chromosome 7 risk locus to *GPNMB*, establishing it as a genetic risk factor for PD [69,70,71]. In our analysis, brain-specific Mendelian randomisation results further supported a causal role for *GPNMB* in PD. Mechanistically, in *GBA1*-related and sporadic PD, lysosomal dysfunction and α-synuclein accumulation trigger microglial activation and induce GPNMB expression [65,69,72,73]. Because *GPNMB* itself can modulate the microglial phenotype, promote phagocytic clearance, and influence α-synuclein processing, its genetic association, disease-specific upregulation, and biomarker profile in GD together point to its involvement in a shared lysosomal–neuroinflammatory axis that connects *GBA1*-linked metabolic defects with PD neurodegeneration.

The *GIPR* gene, which encodes the gastric inhibitory polypeptide receptor, provides an endocrine and metabolic axis linking peripheral metabolic regulation to central neurodegenerative processes [74,75]. As the receptor for the incretin hormone gastric inhibitory polypeptide, the *GIPR* gene product plays a central role in glucose and insulin homeostasis, pancreatic beta-cell survival, and energy balance. In the brain, expression of the GIPR gene has been reported in regions relevant to PD’s pathology, including the hippocampus, substantia nigra, and cortex, where it influences neuronal survival pathways, synaptic plasticity, and microglial activation [75]. Preclinical models of PD indicate that activation of the gastric inhibitory polypeptide receptor, either alone or in combination with stimulation of the glucagon-like peptide 1 receptor (GLP-1), reduces α-synuclein aggregation, protects dopaminergic neurons, and suppresses neuroinflammatory responses, potentially through cyclic adenosine monophosphate protein kinase A (cAMP) response element-binding protein and phosphatidylinositol 3 kinase protein kinase B signalling (PI3K-Akt) pathways [76,77]. Early-stage clinical studies of dual GLP-1 and gastric inhibitory polypeptide receptor agonists in neurodegenerative disease further support the relevance of *GIPR* gene [76]. Within this framework, the *GIPR* gene aligns with glucose and insulin as well as hormonal signalling pathways, providing a mechanistic bridge between systemic metabolic status and neuroprotective processes linked to PD.

Evidence directly linking *FAM89A* and *TESMIN* to PD remains limited. *FAM89A* encodes a protein of largely unknown function, with its highest expression reported in the placenta and adipose tissue [78]. In a proteomic study of absence epilepsy in rats, FAM89A was among the thalamic proteins differing between groups; however, the main finding concerned age-related changes in the thalamic proteome overall, rather than a specific role for *FAM89A* or a defined neuronal function [78]. *FAM89A* expression is upregulated during bacterial infection and helps distinguish bacterial from viral causes [79,80]. Abnormal methylation of *FAM89A* has also been reported in glioma, and the gene shows prenatal relevance [81]. These findings align with the immune metabolic and developmental themes emerging from our analyses, although Parkinson’s disease-focused evidence for FAM89A remains sparse.

*TESMIN* (also known as *MTL5*) encodes a testis-enriched metallothionein-like protein with high affinity for divalent metals such as zinc and copper and an established role in spermatogenesis [82,83]. Its metallothionein-like properties suggest a role in metal buffering and defence against oxidative stress, consistent with the wider biology of metallothioneins [84]. Although no direct experimental or genetic link to PD has been reported, dysregulation of metal ion homeostasis is a recognised feature of its pathogenesis [85]. Lysosomes are central regulators of intracellular metal pools, including iron, zinc, and copper, and their dysfunction disrupts metal homeostasis, precipitating mitochondrial impairment and broader metabolic disturbances [83,86]. Such lysosomal impairment is a hallmark of *GBA1*-associated Parkinson’s disease and is also implicated in sporadic disease, where it drives oxidative stress, perturbs enzyme activity, and promotes α-synuclein aggregation [87,88].

Brain-specific Mendelian randomisation findings provide further insights into plausible mechanisms. The *AGT* gene, encoding angiotensinogen, implicates the renin–angiotensin system in oxidative stress, neuroinflammation, and dopaminergic modulation, a pathway increasingly linked to PD. Expression of AGT initiates a cascade in which angiotensin II, the product of the renin–angiotensin system, binds to type 1 angiotensin receptors in both neurons and microglia under conditions of impaired dopaminergic function [89,90,91,92]. This activation enhances nicotinamide adenine dinucleotide phosphate (NADPH) oxidase activity, increasing reactive oxygen species production, fuelling neuroinflammation, and promoting α-synuclein aggregation within the nigrostriatal system [90,93,94]. In parallel, angiotensin II-mediated signalling affects dopamine release and exerts allosteric inhibition of dopamine type 1 receptors, thereby impairing dopaminergic neurotransmission, an effect that may exacerbate synaptic dysfunction [89,95,96].

Viewed from complementary perspectives on PD’s pathogenesis, the whole-blood Mendelian randomisation findings suggest independent routes through which genetically driven differences in gene expression may influence risk. Genes involved in lipid and cholesterol metabolism, including *LPL* (encoding lipoprotein lipase, which regulates triglyceride hydrolysis) [97] and *METTL7A* (encoding a methyltransferase linked to lipid droplet dynamics) [98,99,100], were highlighted. The *MMP9* gene (encoding matrix metalloproteinase 9, a key mediator of extracellular matrix remodelling) also emerged, linking matrix remodelling to lipid-mediated inflammation. These candidates support mechanisms that may favour α-synuclein misfolding and microglial stress. A complimentary endocrine–metabolic component was represented by *PID1* (encoding phosphotyrosine interaction domain-containing protein 1, a regulator of insulin signalling) [101], together with *GIPR* (encoding the receptor for glucose-dependent insulinotropic polypeptide, an incretin hormone) [76] and *LPL*, implicating insulin resistance and impaired bioenergetics. Neuronal signalling and trafficking candidates included *NECAB2* (encoding neuronal calcium-binding protein 2, involved in endosomal calcium handling) [102] and *RASGRF2* (encoding Ras protein-specific guanine nucleotide-releasing factor 2, a regulator of Ras ERK signalling and synaptic plasticity) [103,104], pointing to corticostriatal dysfunction. Innate immune and extracellular matrix pathways were reflected by *TRIM22* (encoding tripartite motif-containing protein 22, a modulator of interferon responses) [105] and *MMP9* [106], while *NFE2L3* (also known as *NRF3*, encoding nuclear factor erythroid 2-related factor 3, a transcriptional regulator of oxidative stress responses) highlighted antioxidant defences [107]. These dysregulated pathways explain why the risk genes are plausible causal contributors to PD: each anchors a recognised pathway with mechanistic links to dopaminergic vulnerability. Nonetheless, the signals remain hypothesis-generating and warrant experimental validation in relevant cell types and longitudinal cohorts.

Building on the Mendelian randomisation signals, the next step was to test whether they appear in real-world expression data and whether they perturb core metabolic circuits in dopaminergic neurons. Across independent transcriptomic datasets, discriminatory power was concentrated in a small subset of genes rather than the full panel, and the top genes differed by subtype. This points to heterogeneity: instead of one universal gene signature, there seem to be two routes to PD risk. One route is *GBA1*-sensitised, with effects strongest when *GBA1* is deficient and biology leaning towards lysosomal–lipid and redox processes; the other appears to be *GBA1*-independent, centred on neuronal signalling and endocrine–metabolic control. Metabolic modelling further highlighted shared pressure points, including inositol phosphate signalling, glycerophospholipid turnover, glycolysis/gluconeogenesis, and catecholamine handling, while revealing subtype-specific weighting, with greater involvement of fatty acid and bile acid metabolism under *GBA1* deficiency and additional perturbations in nucleotide, one-carbon, and sterol metabolism in sporadic PD. These signatures align with the functional portfolios of the risk genes (lipid/sterol handling, insulin–energy coupling, synaptic/trafficking control, and oxidative–inflammatory responses) and offer a mechanism for how modest expression differences could yield outsized phenotypic effects.

Collectively, these findings delineate a coherent pathophysiological framework with translational relevance. The *GBA1*–lysosomal–lipid/redox axis supports therapeutic strategies aimed at restoring lysosomal competence and redox balance. Interventions such as enzyme replacement and pharmacological chaperones that enhance β-glucocerebrosidase activity, small molecules that stabilise lysosomal membranes, and activators of lysosomal biogenesis could attenuate disease progression [108,109,110]. The identification of GPNMB as a shared lysosomal–neuroinflammatory mediator highlights the potential of targeting microglial activation and *GPNMB*-related signalling to modulate neuroinflammation [69,72,73]. In parallel, the neuronal–endocrine–metabolic axis underscores the therapeutic relevance of metabolic and hormonal modulation. The roles of *GIPR*, *PID1*, and *LPL* implicate incretin and insulin pathways, supporting the growing interest in dual GLP-1/GIP receptor agonists for neuroprotection [74,76,77]. Overall, the integrated genetic, transcriptomic, and metabolic evidence links defined molecular perturbations to plausible treatment avenues, providing a mechanistic bridge between pathophysiology and therapeutic development in both GBA1-related and sporadic Parkinson’s disease.

### Limitations

Several limitations should be considered when interpreting these findings. First, although Mendelian randomisation mitigates confounding and reverse causation, residual horizontal pleiotropy cannot be completely excluded, despite consistent results across multiple Mendelian randomisation methods and non-significant Egger intercepts. Second, the use of bulk-tissue eQTL datasets for instrument selection may mask cell-type-specific effects, particularly within heterogeneous brain regions where the regulatory architecture differs between neurons and glia. Third, our transcriptomic comparisons relied on publicly available datasets with varying sample sizes and normalisation protocols, which may have introduced batch-related heterogeneity despite our use of independent differential expression analyses. Finally, as this study was based solely on integrative computational analyses, experimental validation (e.g., qPCR or Western blot) of the prioritised genes and pathways will be required to confirm their mechanistic roles and diagnostic relevance.

## 5. Conclusions

Convergent genetic, transcriptomic, and metabolic evidence supports a dual-axis model of PD risk, comprising *GBA1*-sensitised lysosomal–lipid/redox pathways and *GBA1*-independent neuronal/endocrine routes. This framework reconciles incomplete penetrance among *GBA1* carriers and explains why different markers dominate across clinical backgrounds. It also offers a practical blueprint for next steps: develop stratified biomarker panels that reflect these axes; test gene–*GBA1* interactions in iPSC-derived neurons, microglia, and peripheral immune cells; and perform targeted perturbations of lipid/redox versus neuronal/endocrine nodes in model systems and longitudinal cohorts. While Mendelian randomisation estimates, genetically proxied expression, and constraint-based models simplify neuronal physiology, the convergence across methods argues that these candidates merit focused validation. In the short term, integrating Mendelian randomisation-prioritised markers into prospective cohorts could refine risk prediction for *GBA1* carriers; in the longer term, mechanistic dissection of the two axes may enable pathway-specific interventions, combining lysosomal–lipid/redox modulation with approaches aimed at synaptic and metabolic resilience. Clinically, such stratification could inform trials by enriching for those most likely to benefit from each route.

## Figures and Tables

**Table 1:** Transcriptomic datasets used to identify shared DEGs in Gaucher disease and Parkinson’s disease.

Disease	Dataset	Cell Type	Data Type	Reference
	GSE183484	iPSCs-macrophages	Expression profiling by array	[47]
	GSE41243	iPSCs-fibroblasts	Expression profiling by array	[48]
	GSE21899	iPSCs-fibroblasts	Expression profiling by array	-
Gaucher disease	GSE263252	Primary fibroblasts	Expression profiling by high-throughput sequencing	[49]
	GSE124283	Primary fibroblasts	Expression profiling by array	[50]
	GSE13675	Chemical-induced MSCs	Expression profiling by array	-
	GSE118511	iPSCs neurons	Expression profiling by high-throughput sequencing	[51]
	GSE78152	iPSC neurons	Expression profiling by array	[52]
	GSE62642	iPSC neurons	Expression profiling by array	[45]
	GSE53424	iPSC neurons	Expression profiling by array	[53]
	GSE24378	iPSC neurons	Expression profiling by high-throughput sequencing	[54]
Parkinson’s Disease	GSE208781	iPSC organoids	Expression profiling by high-throughput sequencing	[55]
	GSE99142	iPSC fibroblasts	Expression profiling by array	-
	GSE184956	Macrophages	Expression profiling by high-throughput sequencing	[56]
	GSE205450	Post-mortem brain tissue	Expression profiling by high-throughput sequencing	[42]
	GSE169755	Post-mortem brain tissue	Expression profiling by high-throughput sequencing	[57]
	GSE20186	Post-mortem brain tissue	Expression profiling by high-throughput sequencing	[54]

## Data Availability

All data generated or analysed during this study are included in this published article and its Supplementary Materials. All of the code generating the results in this article is available upon request.

## Supplementary Materials

The following supporting information can be downloaded at https://www.mdpi.com/article/10.3390/biomedicines13112799/s1, Table S1: Total_export_brainQTL; Table S2: Total_export_plasmaQTL; Table S3: heterogeneity_brainQT; Table S4: heterogeneity_plasmaQT; Table S5: pleiotropy_brainQTL; Table S6: pleiotropy_plasmaQTL; Table S7: sensitivity_brainQTL; Table S8: sensitivity_plasmaQTL.

## Abbreviations

The following abbreviations are used in this manuscript:

GD	Gaucher disease
PD	Parkinson’s disease
DEGs	Differentially expressed genes
iPSC	Induced pluripotent stem cell
eQTL	Expression quantitative trait loci
GWAS	Genome-wide association study
SNPs	Single-nucleotide polymorphisms
ROC	Receiver operating characteristic
AUC	Area under the curve
LASSO	Least Absolute Shrinkage and Selection Operator
FDR	False discovery rate
